# Supplementary figures and images for: Efficacy and Safety of the Combination of Superoxide Dismutase, Alpha Lipoic Acid, Vitamin B12, and Carnitine for 12 Months in Patients with Diabetic Neuropathy
Source: Nutrients. 2020 Oct 23;12(11):3254. doi: 10.3390/nu12113254 (PMC7690794; doi:10.3390/nu12113254)

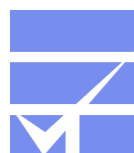

## CONSORT 2010 Flow Diagram

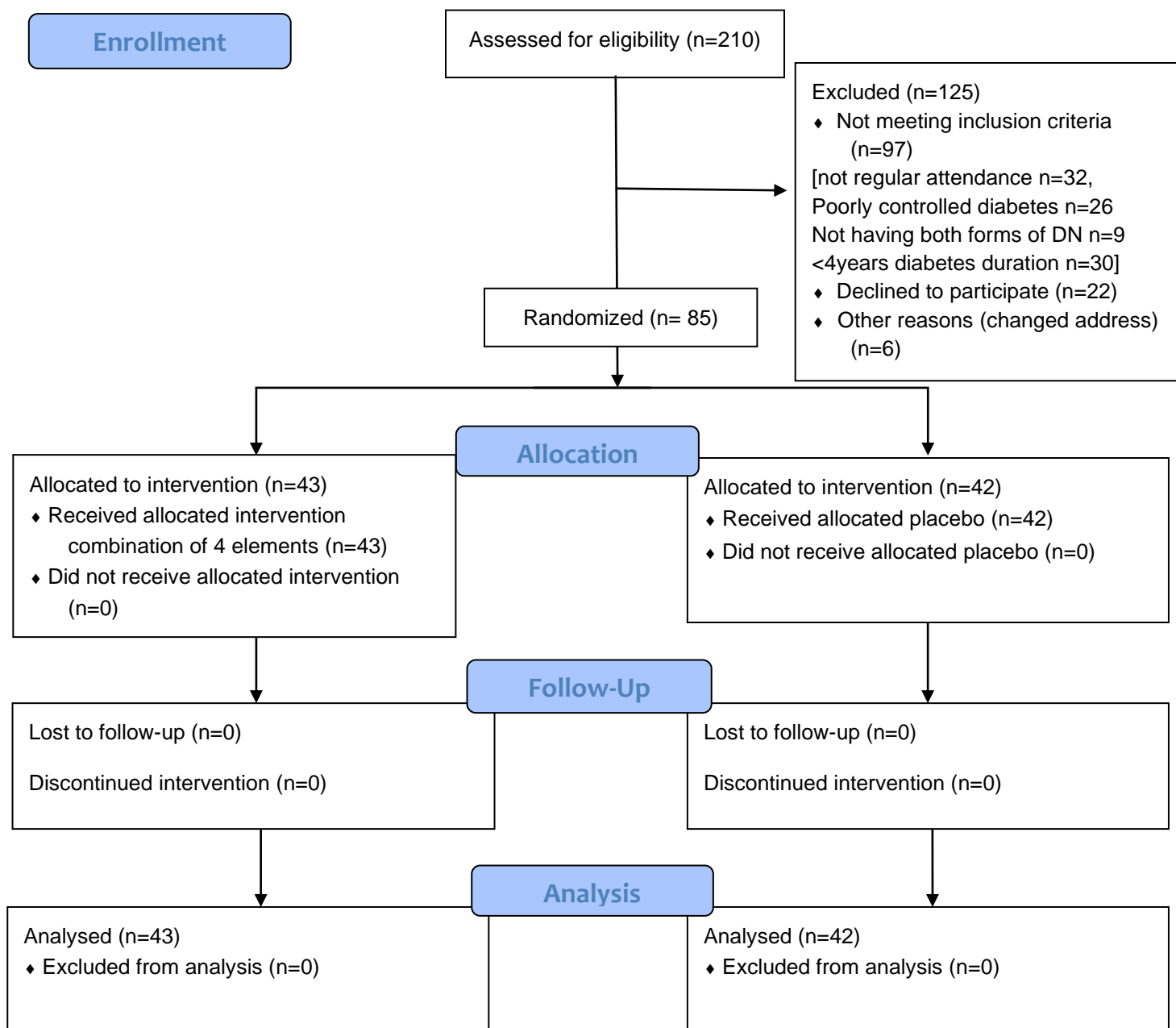

Supplement: Supplementary file 1 [file nutrients-12-03254-s001.pdf]
